# Rapid discrimination of *Bifidobacterium longum* subspecies based on MALDI-TOF MS and machine learning

**DOI:** 10.3389/fmicb.2023.1297451

**Published:** 2023-12-04

**Authors:** Kexin Liu, Yajie Wang, Minlei Zhao, Gaogao Xue, Ailan Wang, Weijie Wang, Lida Xu, Jianguo Chen

**Affiliations:** ^1^College of Life Science, North China University of Science and Technology, Tangshan, China; ^2^Beijing Hotgen Biotechnology Inc., Beijing, China; ^3^Department of Clinical Laboratory, Beijing Ditan Hospital, Capital Medical, Beijing, China; ^4^Beijing YuGen Pharmaceutical Co., Ltd., Beijing, China

**Keywords:** *Bifidobacterium longum* subspecies, MALDI-TOF MS, machine learning, identification, *B. longum*, *B. infantis*

## Abstract

Although MALDI-TOF mass spectrometry (MS) is widely known as a rapid and cost-effective reference method for identifying microorganisms, its commercial databases face limitations in accurately distinguishing specific subspecies of *Bifidobacterium*. This study aimed to explore the potential of MALDI-TOF MS protein profiles, coupled with prediction methods, to differentiate between *Bifidobacterium longum subsp. infantis* (*B. infantis*) and *Bifidobacterium longum subsp. longum* (*B. longum*). The investigation involved the analysis of mass spectra of 59 *B. longum* strains and 41 *B. infantis* strains, leading to the identification of five distinct biomarker peaks, specifically at m/z 2,929, 4,408, 5,381, 5,394, and 8,817, using Recurrent Feature Elimination (RFE). To facilate classification between *B. longum* and *B. infantis* based on the mass spectra, machine learning models were developed, employing algorithms such as logistic regression (LR), random forest (RF), and support vector machine (SVM). The evaluation of the mass spectrometry data showed that the RF model exhibited the highest performace, boasting an impressive AUC of 0.984. This model outperformed other algorithms in terms of accuracy and sensitivity. Furthermore, when employing a voting mechanism on multi-mass spectrometry data for strain identificaton, the RF model achieved the highest accuracy of 96.67%. The outcomes of this research hold the significant potential for commercial applications, enabling the rapid and precise discrimination of *B. longum* and *B. infantis* using MALDI-TOF MS in conjunction with machine learning. Additionally, the approach proposed in this study carries substantial implications across various industries, such as probiotics and pharmaceuticals, where the precise differentiation of specific subspecies is essential for product development and quality control.

## 1 Introduction

*Bifidobacterium longum subsp. infantis* (*B. infantis*) and *Bifidobacterium longum subsp. longum* (*B. longum*), the most abundant *Bifidobacterium* species in the intestinal flora of infants, are essential for their immune development. Human breast milk contains a large amount of human milk oligosaccharides (HMOs), which cannot be digested by infant due to a lack of necessary glucosidases. However, the positive effects of HMOs on newborns’ health are attributed to the “beneficial” microorganisms that specialize in metabolizing HMOs. In contrast to *B. longum*, *B. infantis* typically harbors all the genes required for utilizing HMOs (Duar et al., 2020) and can digest various types of HMOs, including 3′-SL, 6′-SL, 2′-FL, 3′-FL, LNnT, and LacNAc (Zhang et al., 2022). The absence of *Bifidobacterium* and HMO utilization genes in the gut microbiota is associated with inflammation and immune imbalances in early life (Henrick et al., 2021). *B. infantis* is commonly found in breastfed infants in countries with a low prevalence of immune-mediated diseases, such as Bangladesh (Vatanen et al., 2022) and Malawi, but is rare in Europe and North America (Casaburi et al., 2021). However, supplementation with *B. infantis* EVC001, by remodelling the gut microbiome of breastfed infants, reduced intestinal inflammation (Henrick et al., 2019), decreased intestinal Th2 and Th17 cytokines and up-regulated IFNβ, favouring immune development in early life (Henrick et al., 2021). Therefore, accurate identification of *B. longum* and *B. infantis* is essential for efficient screening, functional studies and application development of *B. infantis*.

The current methods used to identify *Bifidobacteria* include PCR, SNP, cgMLST, and MALDI-TOF MS. MALDI-TOF MS is particularly advantageous due to its high throughput, fast speed, and low cost, making it widely used for identifying clinical pathogenic microorganisms and general microorganisms (Gato et al., 2021; Heilbronner and Foster, 2021; Wang H. Y. et al., 2022). However, the successful identification of bacteria using MALDI-TOF MS heavily relies on databases that contain spectra of known organisms and most of the biomarker peaks are in the range m/z 2,000–10,000 (Carvalho et al., 2022; Topić Popović et al., 2023). Most commercial databases only identify bacteria at the species level and lack the ability to accurately differentiate closely related subspecies, such as *B. longum* and *B. infantis*. Although six biomarker peaks have been reported to differentiate between *B. longum* and *B. infantis*, these peaks have not been commercially applied due to their high mass peaks (>15,000 m/z) (Sato et al., 2011), low reproducibility, and lack of availability in commercial databases. Recently, machine learning techniques have been used to accurately identify strains that cannot be distinguished using commercial databases by analyzing protein mass spectra obtained through MALDI-TOF MS (Weis et al., 2022; Kim et al., 2023).

Machine learning (ML) technology encompasses various algorithms such as random forest (RF), support vector machines (SVM), logistic regression (LR) and decision trees (DT) (Weis et al., 2020). ML enables rapid and precise identification of species-specific biomarkers from MALDI-TOF MS spectra, which has been widely implemented to analyze microbial signatures and construct classification models. Recently, the combination of MALDI-TOF MS and ML has gained popularity in classifying clinically pathogenic and drug-resistant bacteria, including *Escherichia coli* (van Oosten and Klein, 2020), *Staphylococcus aureus* (Rodríguez-Temporal et al., 2022), *Klebsiella pneumoniae* (Yu et al., 2023), *Brucella melitensis* (Dematheis et al., 2022), and *Campylobacter* spp. (Feucherolles et al., 2021). However, there is a lack of identification schemes for *Bifidobacterium* subspecies within a specific taxon in these studies. Hence, there is an urgent need to develop a combined machine learning and MALDI-TOF MS method for rapid and accurate identification of *Bifidobacterium* subspecies.

**Figure 1:**
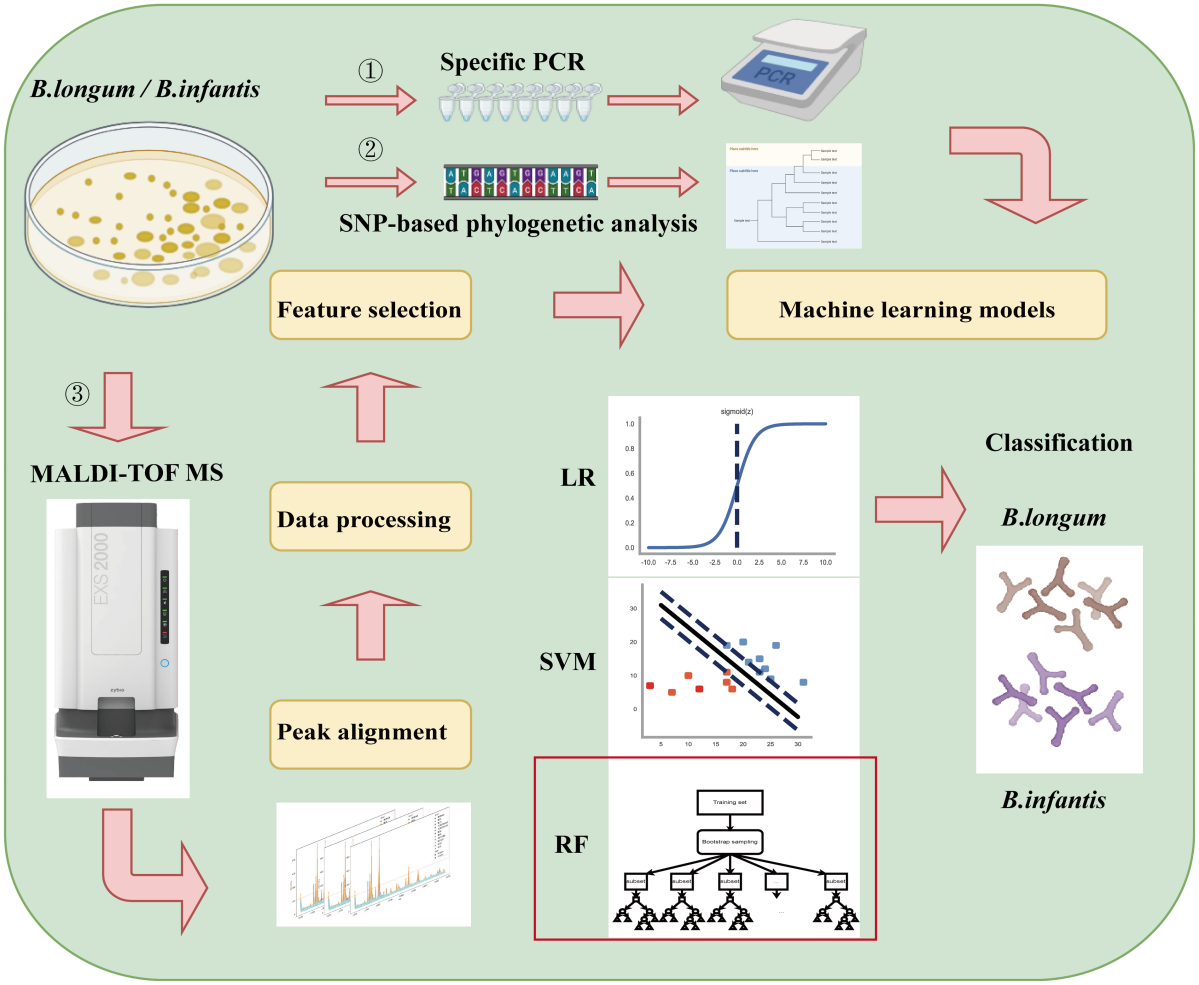
Experimental flow chart of *Bifidobacterium longum* subspecies discrimination based on MALDI-TOF MS and machine learning in this study.

In the present study, we first screened for robust variations in subspecies-specific features between *B. longum* and *B. infantis* based on MALDI-TOF MS analysis and a combination of machine learning methods such as LR, SVM, and RF (Figure 1). The objective of this research was to develop a fast classification tool using Machine-learning-combined MALDI-TOF MS to accurately distinguish between *B. longum* and *B. infantis*.

## 2 Results

### 2.1 Molecular identification by PCR and phylogenetic analysis

**Figure 2:**
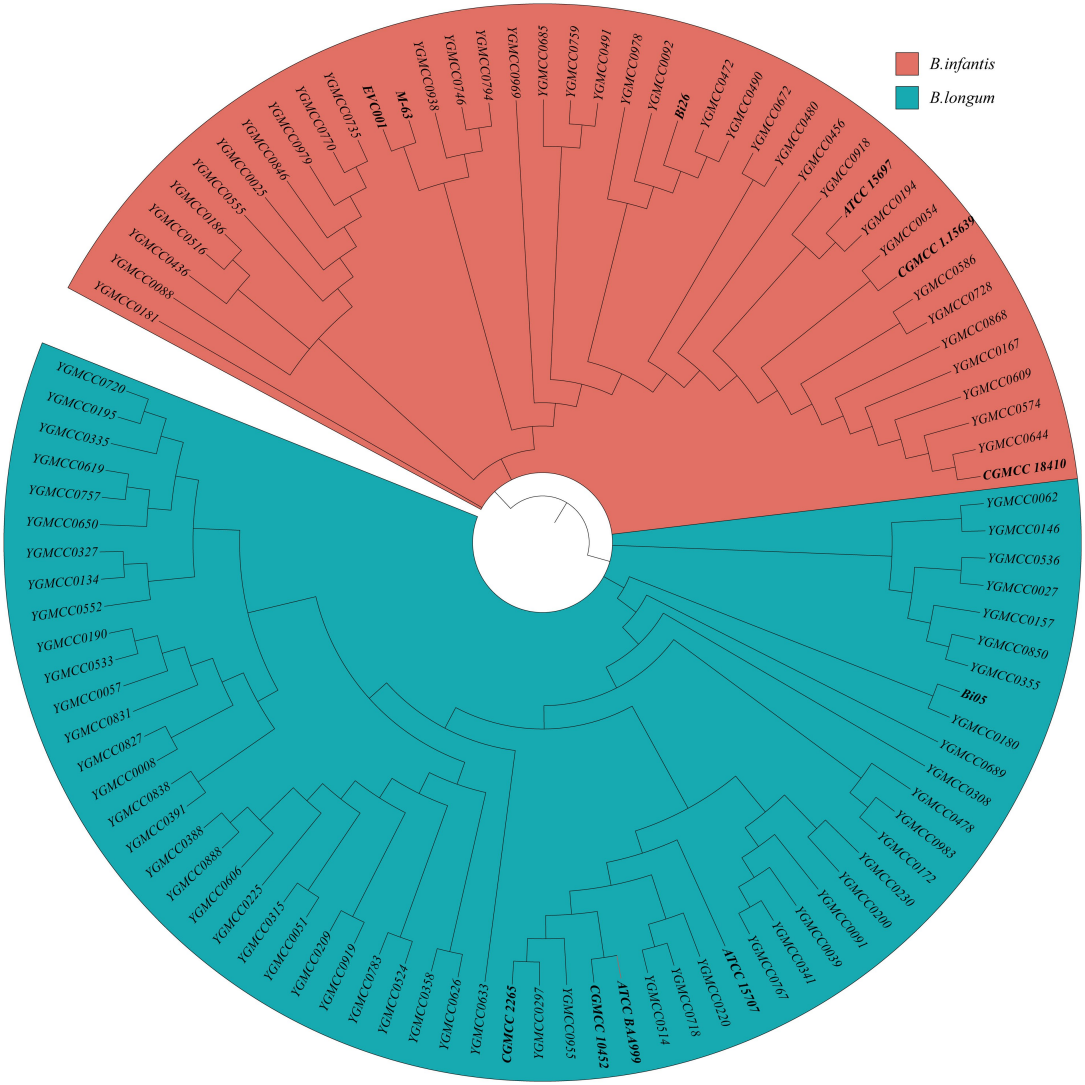
Identification of 41 *B. infantis* strains and 59 *B. longum* strains based on the phylogenetic analysis. The red and blue background represent *B. infantis* or *B. longum* strains, respectively.

Specific primers-based PCR could differentiate between *B. longum* and *B infantis*. Thus, this method was employed to confirm the taxonomic classification of all the strains in study. The specificity and sensitivity of the PCR assay using specific primers for distinguishing the two subspecies were confirmed by successfully differentiation of 11 representative strains. Out of the 89 isolates analyzed, 54 were identified as *B. longum* and 35 were identified as *B. infantis*. For additional confirmation, SNP information obtained from 100 genome sequences were utilized to construct a phylogenetic tree. The tree effectively separated the sequences into two distinct branches. The phylogenetic tree revealed that 59 *B. longum* strains, comprising five typical strains and 54 isolates, clustered together with a blue background, while 41 *B. infantis* strains formed a distinct group with a red background (Figure 2). These findings underscore the efficacy of using phylogenetic tree features for precise classification and identification of *B. longum* and *B. infantis*, which align with the outcomes obtained from specific PCR genotyping (Supplementary Table S1).

### 2.2 Identification of mass spectra for strains

**Figure 3:**
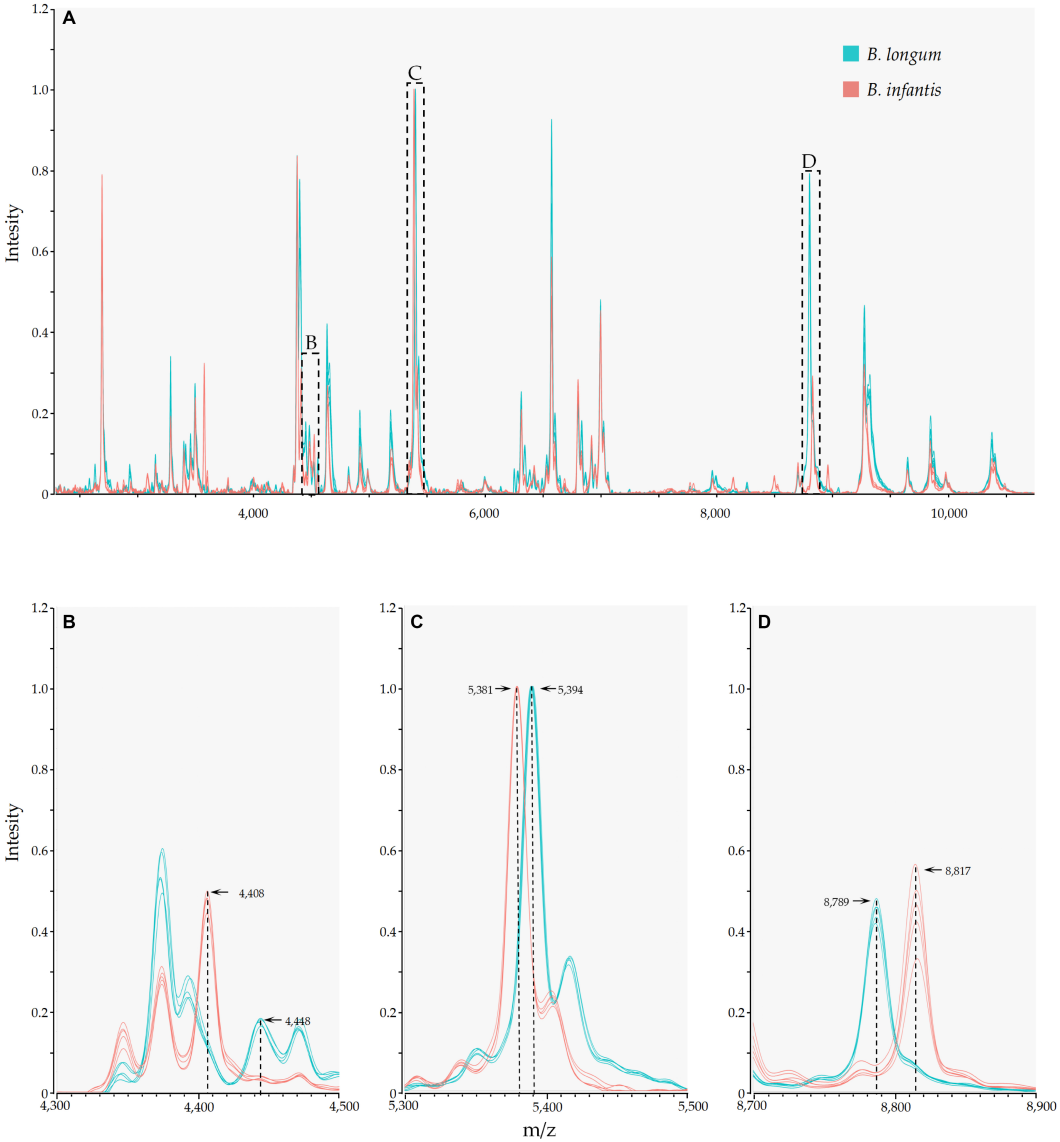
MALDI-TOF MS and species-specific peaks of *B. longum* (Orange) and *B. infantis* (Purple). The y-axis represents the intensity of the peaks, while the x-axis represents the m/z values; **(A)** depicts stowage diagram of *B. longum* and *B. infantis*; **(B−D)** display enlarged views of subspecies-specific peaks as depicted in **A**.

Mass spectrometry results indicated the presence of numerous identical mass spectral peaks for both *B. longum* and *B. infantis*, making accurate differentiation challenging when relying solely on commercial databases (Figure 3A; Supplementary Table S1). However, further analysis unveiled six species-specific peaks that exhibited a high degree of conservation and could serve as potential biomarkers for identification. As shown in Figures 3B–D, peaks at m/z 4448.52 (94.9%, 56/59), 5394.35 (100.0%), and 8789.47 (100.0%) were exclusively found in the spectrogram of *B. longum*. Conversely, peaks at m/z 4408.42 (95.1%, 39/41), 5381.06, and 8817.28 (100.0%) were observed solely in the spectrogram of *B. infantis*. These findings reveal the potential of MALDI-TOF MS to differentiate between *B. longum* and *B. infantis* based on specific peaks with the protein fingerprint profile.

### 2.3 Discovery and identification of protein biomarkers by MALDI-TOF MS

**Figure 4:**
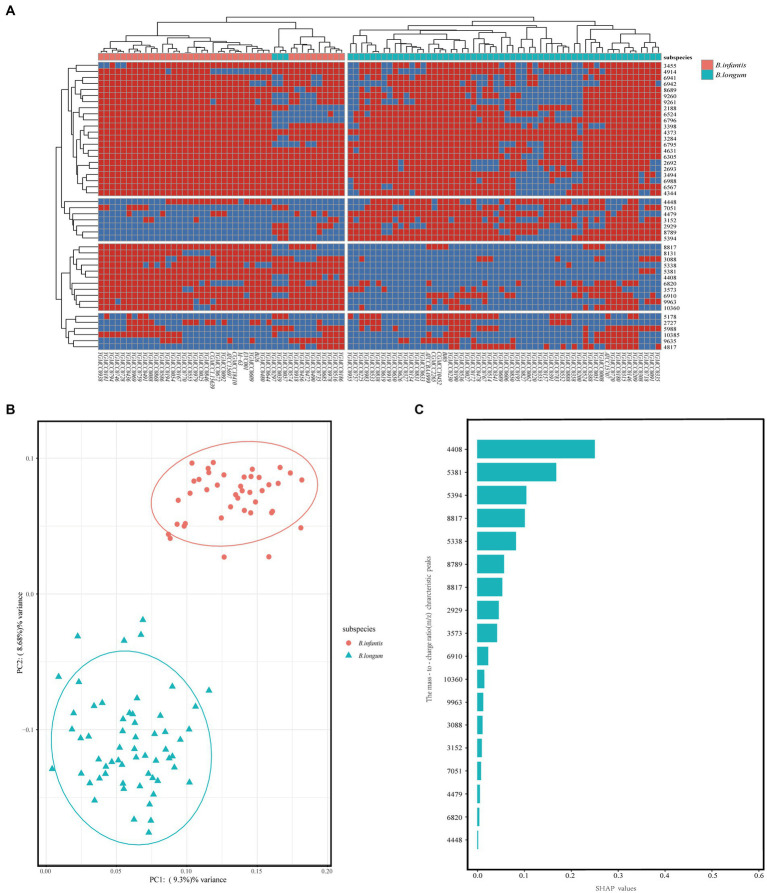
Unsupervised analysis and feature importance evaluation. **(A)** After mass spectral alignment, heat maps were plotted and clustered based on the absence/presence of common characteristic peaks in the top 50% of effective *p* values within subspecies. **(B)** PCA of *Bifidobacterium longum* subsp. Each dot on the PCA plot represents the average spectrum of each strain, blue for *B. longum,* and red for *B. infantis*. **(C)** Assessment of feature importance in a RF model for distinguishing between *B. longum* and *B. infantis*.

To investigate the applicability of MALDI-TOF MS for discriminating *B. longum* and *B. infantis*, we performed redundant removal, smoothing, and alignment of 400 spectra from 100 strains using OpenMS software. We identified some potential characteristic peaks and constructed a mass spectrometry data matrix for further analysis. To further investigate the distinguishing features, we performed a more specific heatmap clustering analysis of the mass spectrometry data matrix (Figure 4A). Then we performed the principal component analysis (PCA) of the mass spectrum data matrix obtained from the above method. The PCA plot clearly showed the distinct clustering patterns of the two subspecies (Figure 4B), indicating their potential for differentiation. Finally, 18 potential discriminatory peaks were identified, with 11 peaks specific to the *B. infantis*, including the 3,088 m/z, 3,573 m/z, 4,408 m/z, 5,338 m/z, 5,381 m/z, 6,820 m/z, 6,910 m/z, 8,131 m/z, 8,817 m/z, 9,963 m/z, 10,360 m/z. *B. longum* with seven specific peaks, respectively, are located at the 2,929 m/z, 3,152 m/z, 4,448 m/z, 4,479 m/z, 5,394 m/z, 7,051 m/z, 8,789 m/z. These discriminatory peaks are expected to serve as potential features for constructing the classifiers. Furthermore, to assess the importance of features, we analyzed between 18 feature peaks and drew bar graphs (Figure 4C) and found higher SHAP values for feature peaks at 4408 m/z, 5,381 m/z, 5,394 m/z and 8,817 m/z. This suggests that these peaks seem particularly well suited for building classifiers.

To gain insights into the identities of these characteristic peaks, we conducted a comparison between the experimental m/z values and genomic data. This analysis suggested that the ion peaks at m/z 5,381 and 5,394 corresponded to the 50S ribosomal protein L34. Additionally, peaks at m/z 8,817 and 7,051 were associated with 50S ribosomal proteins L27 and L30, respectively. The peak at 4408 m/z indicated the presence of the 30S ribosomal protein S5. Moreover, we identified matches with proteins from the DUF (domain of unknown function) family, including m/z 4,479, 8,789, and 9,963. Proteins belonging to the DUF family are characterized by a conserved EYA motif and a length ranging from 66 to 95 amino acids. However, their functional roles remain elusive due to the lack of annotation.

**Figure 5:**
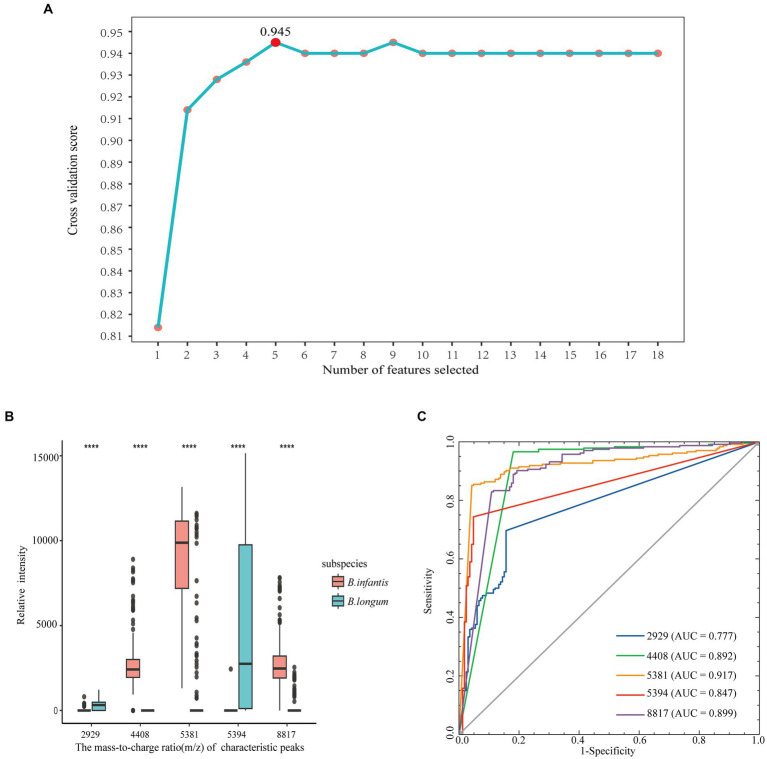
Recursive feature elimination. Line plot of 18 characteristic peaks and cross-validation fractions after REFCV **(A)**, and boxplot of mass-to-charge ratio and relative intensity of 5 optimal characteristic peaks between the two subspecies of *Bifidobacterium longum* (**** represents the *p* value of the difference < 0.0001). **(B)**. ROC curve and AUC value of the five optimal characteristic peaks **(C)**.

The 18 feature peaks obtained above were conducted recursive feature elimination using a logistic regression algorithm with cross-validation to determine the optimal feature set. Figure 5A illustrated that the highest cross-validation score of 0.945 was achieved when using five features. These five optimal features include m/z 2,929, 4,408, 5,381, 5,394, and 8,817. Among them, m/z 2,929 and 5,394 were characteristic peaks of *B. longum*, while the remaining peaks were specific to *B. infantis*. The significance of the five selected features was presented using a boxplot (Figure 5B), and the results indicated that the *p*-values of the five features, as determined by Fisher’s exact test, were all less than 0.001. In addition, individual ROC curves were plotted for the five selected features (Figure 5C). The AUC values ranged from 0.777 for m/z 2,929 to 0.917 for m/z 5,381. It indicates that the features obtained after recursive elimination can contribute to achieving the best classification performance.

### 2.4 Construction of the machine learning models

We developed three commonly used machine learning models: LR, SVM, and RF, for microbial discrimination. The dataset utilized for model construction consisted of 100 strains, with their subspecies verified through PCR and phylogenetic analysis. This dataset was randomly divided into a training set for building the models and a test set for evaluation their performance. Based on the results obtained from the test set, we calculated performance metrics such as sensitivity, specificity, accuracy, Youden coefficient, and AUC value (see Table 1).

**Table 1:** Frequencies and assignments of species-specific peaks for *B. longum* and *B. infantis.*

Experimental m/z	Presence of peak (%)		Theoretical m/z	Possible presence of protein
	*B. longum*	*B. infantis*		
2,929	77.97 (46/59)	7.32 (3/41)	2,932	Hypothetical protein
3,088	23.72 (14/59)	82.93 (34/41)	3,088	NAD(P)-binding domain-containing protein
3,152	69.49 (41/59)	12.20 (5/41)	3,150	Integrase partial
3,573	30.50 (18/59)	95.12 (39/41)	3,573	Restriction endonuclease
4,408	0.00 (0/59)	95.12 (39/41)	4,406	30S ribosomal protein S5 partial
4,448	55.93 (33/59)	29.27 (12/41)	4,447	50S ribosomal protein L9 partial
4,479	74.58 (44/59)	19.51 (8/41)	4,480	DUF600 family protein partial
5,338	6.78 (4/59)	80.49 (33/41)	5,338	Permease
5,381	10.17 (6/59)	100.0 (41/41)	5,377	50S ribosomal
5,394	81.36 (48/59)	0.00 (0/41)	5,391	Protein L34
6,820	28.81 (17/59)	78.05 (32/41)	6,822	Transporter drug/metabolite exporter family
6,910	38.98 (23/59)	97.56 (40/41)	6,910	Transposase
7,051	67.80 (40/59)	14.63 (6/41)	7,051	50S ribosomal protein L30
8,131	0.00 (0/59)	63.41 (26/41)	8,135	IS3 family transposase partial
8,817	13.56 (8/59)	87.80 (36/41)	8,816	50S ribosomal protein L27
8,789	79.66 (47/59)	2.44 (1/41)	8,789	DUF905 domain-containing protein
9,963	28.81 (17/59)	92.68 (38/41)	9,963	DUF4244 domain-containing protein
10,360	30.50 (18/59)	92.68 (38/41)	10,364	50S ribosomal protein L13 partial

The classification performance parameters of the three models are shown in Table 2. Among them, RF achieved the highest accuracy, AUC, and Youden coefficient, all equal to 1.0, indicating its superior ability to discriminate between the two subspecies. The sensitivity of all three models was 1.0, which means that they could correctly identify all the positive cases. The RF model demonstrated the highest specificity with a value of 1.0, whereas the LR and SVM models exhibited a specificity of 0.931. The RF model also has the highest AUC value of 1.0, demonstrating excellent classification performance. The SVM model’s AUC was slightly better than that of the LR model, with values of 0.995, and 0.993, respectively. The Youden coefficient, reflecting the overall efficiency of the RF model, was 1.0, while for the SVM and LR models, it was 0.931.

**Table 2:** Model result metrics for three machine learning models in validation dataset.

Machine learning models	Specificity	Sensibility	Youden	AUC	Accuracy
LR	0.931	1.000	0.931	0.993	0.958
SVM	0.931	1.000	0.931	0.995	0.958
RF	1.000	1.000	1.000	1.000	1.000

### 2.5 Assessment of practical application of the machine learning model

An external dataset comprising 240 spectra obtained from 60 *Bifidobacterium longum* strains was collected. These isolates were obtained under identical experimental conditions. To validate the model’s effectiveness, the three trained models were utilized to predict the subspecies of these 60 strains.

**Figure 6:**
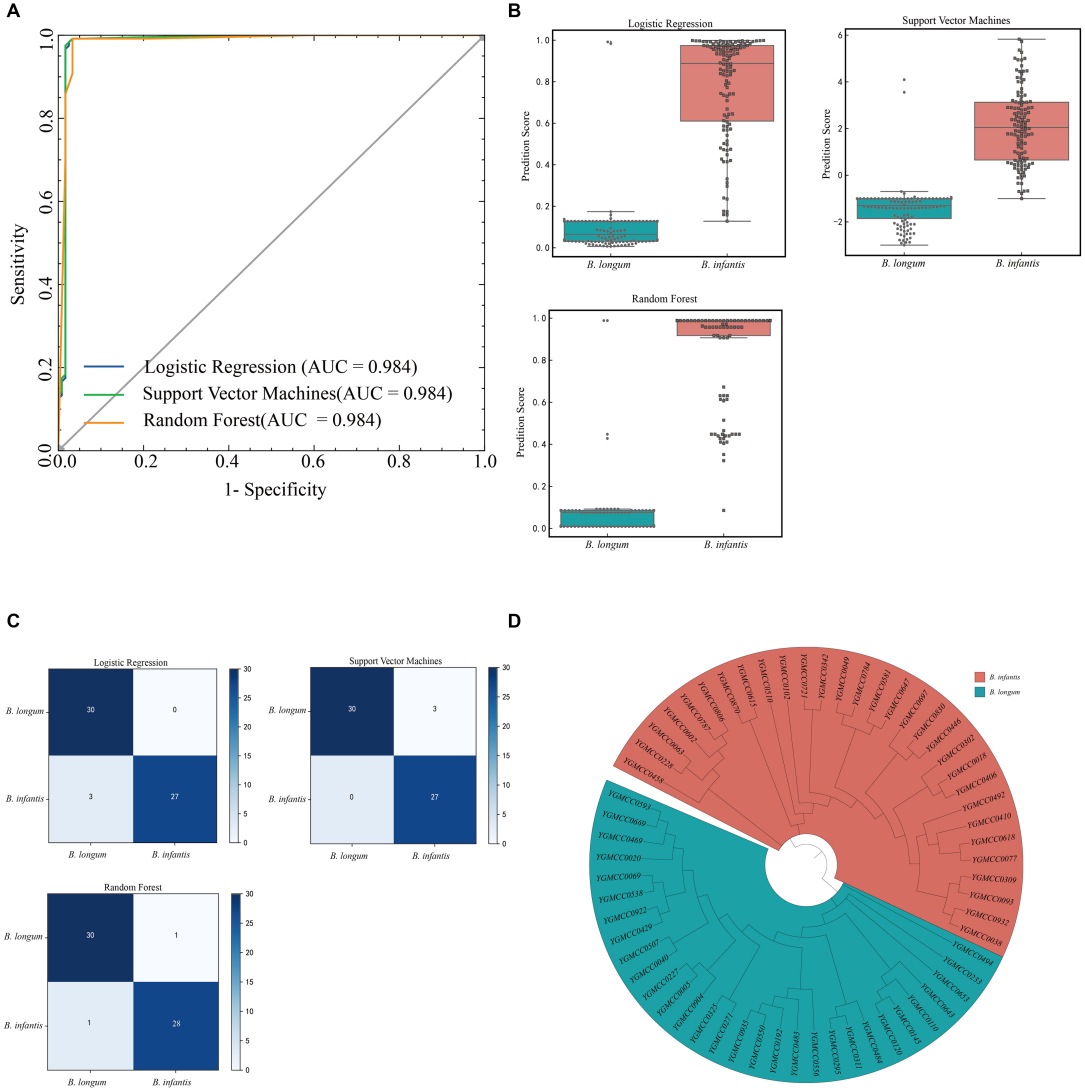
Model evaluation in test dataset. **(A)** ROC curves and AUC of three machine learning models in the test dataset. **(B)** Boxplot of the verification score of the three machine learning models on the spectral data of the external test set. **(C)** Confusion matrix of external strain identification results for three models. **(D)** Cluster analysis of isolated *B. infantis* or *B. longum* strains used for external validation set based on phylogenetic analysis. The red and blue background represnt *B. infantis* or *B. longum* strains, respectively.

Among the three models, both LR and SVM model exhibited a specificity of 0.983, while it was 0.967 for the RF model. However, the LR model demonstrated a higher sensitivity (0.942) compared to the SVM model (0.883) and the RF model (0.900). Regarding accuracy, the RF model outperformed the SVM model and the LR model, achieving an accuracy rate of 0.954. To provide a more intuitive comparison of the models performance, we plotted the ROC curve (Figure 6A) and calculate the AUC values. All three models exhibited very similar AUC values, accurately measured at 0.984. The RF model had the highest Youden index (0.908), surpassing the SVM model (0.867) and the LR model (0.883). Figure 6B illustrated the distribution of prediction scores indicating the likelihood of being *B. infantis* strains for the two subspecies, as determined by the three models.

Based on the four data points results, we established the prediction conditions for the strain subspecies model. A confusion matrix for external strain identification was calculated based on the voting results (Figure 6C). Specific PCR test results and phylogenetic analysis results (Figure 6D) showed consistency. The results from specific PCR tests and phylogenetic analysis (Figure 6D) were consistent with these findings (Supplementary Table S1). Among them, in the LR model, the identification of *B. longum* was in line with PCR and phylogenetic results. However, for the *B. infantis*, specifically YGMCC0271, YGMCC0192, and YGMCC0550, there was inconsistency, with an accuracy of 95%. In the SVM model, the identification of YGMCC0618, YGMCC0063, and YGMCC0038 did not align with PCR and phylogenetic results, resulting in an accuracy rate of 95%. Lastly, in the RF model, the identification of YGMCC0063 and YGMCC0120 differed from PCR and SNP results, achieving an accuracy rate of 96.67%. Based on the external strain identification results, the RF model emerged as the optimal choice.

## 3 Discussion

Genome-based taxonomy is a more standard method of classifying microorganisms than traditional methods (Parks et al., 2018). However, it is time-consuming, expensive, and labor-intensive, and fails to meet the demand for rapid and high-throughput identification of microorganisms. In recent years, MALDI-TOF MS has gained increasing importance in clinical microbial taxonomy as a fast, high-throughput, and robust method for microbial identification. It relies on the detection of microbial housekeeping and ribosomal proteins (Kim et al., 2022a; Haider et al., 2023). Nonetheless, while MALDI-TOF MS can identify bacteria at the species level, it struggles to accurately distinguish closely related species or subspecies. Machine learning algorithms have the capability to identify specific information in mass spectrometry data and analyze relationships among different features, enabling more precise analysis (Weis et al., 2020). By combining machine learning with MALDI-TOF MS, it becomes possible to accurately identify closely related microorganisms at the subspecies level (De Bruyne et al., 2011; Rodríguez-Temporal et al., 2023). Recent studies have demonstrated the application of machine learning techniques in overcoming the limitations of mass spectrometry, such as detecting antibiotic-resistant microorganisms (Yoon and Jeong, 2021), analyzing antimicrobial resistance (Feucherolles et al., 2021), and distinguishing closely related species. By utilizing features obtained from MALDI-TOF MS, SVM algorithms have successfully differentiated clinically resistant strains of carbapenem, methicillin, and β-lactam antibiotics, as well as predicted resistance phenotypes with high accuracy (Ho et al., 2017; Wang J. et al., 2022). Furthermore, the combination of MALDI-TOF MS and machine learning is commonly used to distinguish closely related foodborne microorganisms. For example, an SVM-RBF model achieved a prediction accuracy of approximately 100% in accurately identifying *W. cibaria* and *W. confusa* (Kim et al., 2023).

In our research, we have found that distinguishing closely related species using MALDI-TOF MS can be challenging due to the similarities in their protein fingerprints. MALDI-TOF MS generates a report of the ten closest matches for an unknown species based on mass spectra and the consistency of reference strains in the database. However, when different species within the same genus or different subspecies within the same species have high scores among the top ten matches, accurately identifying the microorganism becomes difficult. Previous studies have attempted to distinguish between *Bifidobacterium longum* subspecies (Kim et al., 2022b) and *Bifidobacterium animalis* subspecies (Jahan et al., 2021) using MALDI-TOF MS. However, these studies had limitations in terms of sample size, unsystematic markers, and lack of validation data, and have not been commercially applied. In this study, our focus was specifically on identifying *B. longum* and *B. infantis* using MALDI-TOF MS. We discovered that commercial databases were unable to accurately differentiate between these two subspecies, which aligns with previous findings (Yahiaoui et al., 2020; Jahan et al., 2021; Kim et al., 2022b).

The aim of this study was to evaluate the ability of MALDI-TOF MS combined with machine-learning methods to rapidly and accurately discriminate between the closely related *B. longum* and *B. infantis*. We employed advanced machine learning algorithms and a larger sample size to enhance statistical significance. We ensured systematic biomarker collection and data analysis to improve the reliability and repeatability of our findings. We examined 400 mass spectra from 100 *Bifidobacterium longum* strains and used a logistic regression model with recursive feature elimination to identify the five most significant mass peaks. Among these peaks, the masses at 2929 and 5,394 m/z were specific to *B. longum*, while the masses at 4408, 5381, and 8,817 m/z were specific to *B. infantis*. These mass peaks can potentially serve as biomarkers for distinguishing between these two species. Using these biomarkers, we developed machine learning models employing LR, RF, and SVM algorithms. All three models exhibited excellent performance in identifying the spectrogram, with the RF model demonstrating high accuracy in discriminating between *B. longum* and *B. infantis*. Furthermore, after evaluating mass spectrum identification results through voting, the RF model achieved the highest accuracy in practical strain identification applications (see Table 3).

**Table 3:** Model result parameters for three machine learning models on an test dataset.

Machine learning models	Specificity	Sensibility	Youden	AUC	Accuracy
LR	0.983	0.900	0.883	0.984	0.942
SVM	0.983	0.883	0.867	0.984	0.933
RF	0.967	0.942	0.908	0.984	0.954

## 4 Materials and methods

### 4.1 Bacterial strains

Twelve reference strains and eighty-nine strains of *Bifidobacterium longum* subspecies, isolated at Beijing Yujing Pharmaceutical Co., Ltd., were selected to explore potential biomarkers (Table 4). The bifidobacterial strains were incubated for 48 h at 37°C under anaerobic conditions. *E. coli* ATCC 25922 incubated for 24 h at 37°C in Luria-Bertani (Solarbio, Beijing, China) agar was applied to external calibration of MALDI-TOF MS.

**Table 4:** Strain information used in this study.

Bacterial strains		Origins
Reference strains		
*Bifidobacterium longum subsp. longum* (*B. longum*)	ATCC 15707	^1^ATCC
	ATCC BAA999	
	CGMCC 10452	^2^CGMCC
	CGMCC 2265	
	Bi05	^3^IFF
*Bifidobacterium longum subsp. infantis* (*B. infantis*)	ATCC 15697	ATCC
	CGMCC 1.15639	CGMCC
	CGMCC 18410	
	Bi26	IFF
	EVC001	^4^Evolve
	M-63	^5^MORINAGA
*Escherichia coli*	ATCC 25922	ATCC
Isolates (^7^N)		
*Bifidobacterium longum* subspecies (149)		^6^YGMCC

^1^ATCC, American type culture collection;^2^CGMCC, China General Microbiological Culture Collection Center;^3^IFF, International Flavors & Fragrances Inc.;^4^Evolve, Infinant Health™;^5^MORINAGA, Morinaga Milk Industry Co., Ltd.;^6^YGMCC, Beijing Yujing Pharmaceutical Co., Ltd.;^7^N, Number of isolates.

### 4.2 MALDI-TOF MS analysis

Proteins from *B. longum* and *B. infantis* were extracted using the ethanol-formic acid-extraction method (Cuénod et al., 2023). Concisely, fresh bacterial culture was suspended in 300 μL of ddH_2_O to which 900 μL of ethanol was added. The bacterial suspension was centrifuged at high speed (10,000× g) for 2 min, the supernatant was removed to completely discard the residual ethanol and recentrifuged. The resulting pellet was resuspended in 20 μL of 70% formic acid to which an equal volume of acetonitrile was added. After centrifugation at 10,000× g for 2 min, 1 μL of each supernatant was transferred to the 96-position MALDI-TOF target plate, allowed to air dry, and then overlaid with 1 μL of the matrix solution (10 mg/mL of α-cyano-4-hydroxy-cinnamic acid (HCCA) in 50% (v/v) acetonitrile with 2.5% (v/v) trifluoroacetic acid).

The mass spectra were acquired using an EXS2000 MALDI-TOF MS (Zybio Inc., Chongqing, China) equipped with a 200 Hz smart-beam solid-state laser and operated in positive linear mode (Xiong et al., 2023). Mass spectra were automatically recorded within a mass range of 2–20 kDa with a total of 200 laser shots. *E. coli* ATCC 25922 was used for mass calibration and instrument parameter optimization, with an average deviation of molecular weight less than 300 ppm after correction. MS data were analyzed using MDT Master (version 1.1). log scores ≥2.0 were accepted for the identification at the species level, and log scores <2.0 and ≥ 1.7 were used for identification at the genus level or the presumptive species level. Log scores below 1.7 were considered unreliable. For establishing stable machine learning models, four high-quality mass spectra (log scores ≥2.3, stable benchmarks, abundant protein peaks, and uniform distribution) were selected in each strain.

### 4.3 Species identification based on PCR and genomics sequences

For the identification of the isolates, genomic DNA was extracted using Easy Pure Bacteria Genomic DNA Kit (Trans, Beijing, China) in accordance with the manufacturer’s instructions. Then, 1 μL of supernatant was used for the following PCR reaction, the reaction mixture contained 10 μL of SapphireAmp^®^ Fast PCR Master Mix (TaKaRa, Beijing, China), 0.5 μL of each primer (10 μM), 1 μL of DNA template, and 8 μL of ddH2O. Specific primers were listed in Table 5. PCR reactions were conducted as follows: one cycle of initial denaturation at 98°C for 3 min, followed by 35 cycles of 98°C for 10 s, 55°C for 10 s, and 72°C for 5 s, and a final extension at 72°C for 2 min. The PCR products were observed by an Agarose gel imaging system (Tanon, Shanghai, China).

**Table 5:** Specific primer information used in this study.

Target	Primer	Sequence (5′–3′)	Size (bp)
*B. longum*	B.lon_831_F	TTCCAGTTGATCGCATGGTC	831
	B.lon_831_R	GGGAAGCCGTATCTCTACGA	
*B. infantis*	B.inf_832_F	TTCCAGTTGATCGCATGGTC	832
	B.inf_832_R	GGAAACCCCATCTCTGGGAT	

Total 149 unknown *Bifidobacterium longum* strains were cultured anaerobically at 37°C for 24 h, then the cultured liquid (50 mL) was centrifuged at 12,000 × g and 4°C for 10 min to collect the cell biomass. Genomic DNA of 149 unknown *Bifidobacterium longum* strains were extracted using a Wizard^®^ Genomic DNA Purification Kit (Promega, United States). Purified genomic DNA was quantified using a TBS-380 fluorometer (Turner BioSystems Inc., Sunnyvale, CA, United States). High-quality DNA (OD260/280 = 1.8–2.0, ≥10 μg) was used for further research. Genomic DNA was sequenced using Illumina sequencing (Illumina, Inc.). The data generated from Illumina platforms were used for bioinformatics analysis.

The phylogenetic analysis included the comparison of genomic sequences from 5 standard strains of *B. infantis*, 6 standard strains of *B. longum*, and an additional 149 unknown *B. longum* strains from our laboratory. These sequences were compared with the genomic sequence of ASM19655v1, which served as the reference genome. The analysis was performed using the Parsnp software, focusing on the core genome (Treangen et al., 2014; Wang et al., 2023). The iTOL (Interactive Tree of Life) tool was utilized to visualize and explore the phylogenetic tree (Letunic and Bork, 2019; Pereira et al., 2023), facilitating the identification and classification of *B. longum* subspecies based on their phylogenetic positions.

### 4.4 Genomic data mining and identification of biomarker proteins

To investigate the significance of using unique peaks from mass spectrum data as biomarkers, we conducted genomic data mining using publicly available databases. The genome sequences of *B. longum* and *B. infantis* were obtained from the National Center for Biotechnology Information (NCBI) database. To annotate the selected protein biomarkers, the web-based ProtParam tool^1^ was utilized to calculate their theoretical molecular weights based on the translated amino acid sequences. Subsequently, a custom script was employed to filter and align the selected proteins, identifying the most relevant proteins enriched in the vicinity of the characteristic peaks.

### 4.5 Model construction and verification

#### 4.5.1 Data preprocess

The MS data obtained using openMS (v2.8) software exhibited high quality, allowing for alignment of peaks obtained from different batches. The processed peak map data matrix was subjected to PCA to access the potential of the features. In addition, a heatmap was drawn for cluster analysis using the R language (v4.2.2). After obtaining the cluster branches of the potential feature peaks, the importance parameters of the features and evaluate the importance of the features.

The dataset consisting of 400 spectra from 59 *B. longum* and 41 *B. infantis* was randomly divided into 70% training and 30% test datasets. The data of subspecies type was binarized, with 0 representing the long subspecies and 1 representing the infant subspecies. All peaks (features) were scaled using Min-Max scalar to ensure variables at different scales contributed equally to the model fitting process.

#### 4.5.2 Classifier model construction

Firstly, feature selection was carried by a meta-converter approach based on a logistic regression classifier with scikit-learn (v1.3.0). Recursive feature elimination with 5x cross-validation (RFECV) was applied to discard irrelevant features and improve the model’s generalization ability.

Secondly, SHAP (SHapley Additive exPlanations) was used to interpret predictions. SHAP is a unified framework that assigns importance values to each feature for a specific prediction and identifies which feature is most important, facilitating the understanding of a machine learning model’s decision-making process (Lundberg and Lee, 2017).

Thirdly, three machine learning algorithms including random forest (RF), logistic regression (LR), and support vector machine (SVM) were used to construct the distinguishing models using the scikit-learn package. The performances of the models were evaluated by generating the confusion matrix on the test dataset. The ROC curve was plotted using the Matplotlib package, and the area under the subject operating characteristic curve (AUROC) was calculated as a measure of classifier performance. The Youden index was utilized to determine the optimal cutoff threshold and calculate the sensitivity, specificity, and accuracy metrics for the model.

To assess the practical applicability of the model in strain identification, we performed an external validation using a new dataset. Each strain in this dataset was accompanied by four mass spectra collected under identical experimental conditions. Subsequently, we compared the identification outcomes with those obtained through specific PCR detection and phylogenetic analysis.

## 5 Conclusion

In our research, we successfully demonstrated the effectiveness of combining MALDI-TOF-MS with machine learning to accurately discriminate between *B. longum* and *B. infantis*. We identified everything from protein fingerprints to potential biomarkers, and developed three spectral map identification models using the ML algorithm, and finally evaluated the various performance metrics and voted to find the optimal algorithm. The algorithm is highly reliable and accurate in distinguishing the two subspecies. This approach has the potential to be applied in various industries, such as the food or pharmaceutical industry, for rapid and cost-effective identification of *B. longum* and *B. infantis*. Furthermore, the identification strategy presented in this study can also be extended to other closely related species.

## Data availability statement

The names of the repository/repositories and accession number(s) can be found below: NCBI; PRJNA1020989.

## Author contributions

KL: Investigation, Methodology, Validation, Writing – original draft. YW: Investigation, Methodology, Validation, Writing – original draft. MZ: Data curation, Investigation, Methodology, Validation, Writing – original draft. GX: Software, Validation, Writing – original draft. AW: Data curation, Writing – review & editing. WW: Supervision, Validation, Writing – review & editing. LX: Data curation, Methodology, Supervision, Writing – review & editing. JC: Project administration, Supervision, Writing – review & editing.

## Footnotes

^1^https://web.expasy.org/protparam/
