# Supplementary material for: Rapid discrimination of Bifidobacterium longum subspecies based on MALDI-TOF MS and machine learning
Source: Front Microbiol. 2023 Dec 4;14:1297451. doi: 10.3389/fmicb.2023.1297451 (PMC10726008; doi:10.3389/fmicb.2023.1297451)
Supplement: Supplementary file 1 [file Table_1.DOCX]

Supplementary Table 1. Identification of *B. longum* and *B. longum* by MALDI-TOF MS, specific PCR, phylogenetic tree and ML model

| Sample ID | MALDI-TOF MS | | PCR | SNP | MODEL | | |
| --- | --- | --- | --- | --- | --- | --- | --- |
|  | organism(best match) | Score Value |  |  | LR | SVM | RF |
| YGMCC0309 | *Bifidobacterium longum* | 2.411 | *B.longum* | *B.longum* | *B.longum* | *B.longum* | *B.longum* |
| YGMCC0647 | *Bifidobacterium longum* | 2.410 | *B.longum* | *B.longum* | *B.longum* | *B.longum* | *B.longum* |
| YGMCC0581 | *Bifidobacterium longum* | 2.529 | *B.longum* | *B.longum* | *B.longum* | *B.longum* | *B.longum* |
| YGMCC0697 | *Bifidobacterium longum* | 2.304 | *B.longum* | *B.longum* | *B.longum* | *B.longum* | *B.longum* |
| YGMCC0510 | *Bifidobacterium longum* | 2.538 | *B.longum* | *B.longum* | *B.longum* | *B.longum* | *B.longum* |
| YGMCC0932 | *Bifidobacterium longum* | 2.537 | *B.longum* | *B.longum* | *B.longum* | *B.longum* | *B.longum* |
| YGMCC0342 | *Bifidobacterium longum* | 2.322 | *B.longum* | *B.longum* | *B.longum* | *B.longum* | *B.longum* |
| YGMCC0093 | *Bifidobacterium longum* | 2.317 | *B.longum* | *B.longum* | *B.longum* | *B.longum* | *B.longum* |
| YGMCC0077 | *Bifidobacterium longum* | 2.416 | *B.longum* | *B.longum* | *B.longum* | *B.longum* | *B.longum* |
| YGMCC0492 | *Bifidobacterium longum* | 2.595 | *B.longum* | *B.longum* | *B.longum* | *B.longum* | *B.longum* |
| YGMCC0618 | *Bifidobacterium longum* | 2.486 | *B.longum* | *B.longum* | *B.longum* | *B.infantis* | *B.longum* |
| YGMCC0787 | *Bifidobacterium longum* | 2.442 | *B.longum* | *B.longum* | *B.longum* | *B.longum* | *B.longum* |
| YGMCC0806 | *Bifidobacterium longum* | 2.552 | *B.longum* | *B.longum* | *B.longum* | *B.longum* | *B.longum* |
| YGMCC0602 | *Bifidobacterium longum* | 2.309 | *B.longum* | *B.longum* | *B.longum* | *B.longum* | *B.longum* |
| YGMCC0228 | *Bifidobacterium longum* | 2.396 | *B.longum* | *B.longum* | *B.longum* | *B.longum* | *B.longum* |
| YGMCC0063 | *Bifidobacterium longum* | 2.501 | *B.longum* | *B.longum* | *B.longum* | *B.infantis* | *B.infantis* |
| YGMCC0018 | *Bifidobacterium longum* | 2.474 | *B.longum* | *B.longum* | *B.longum* | *B.longum* | *B.longum* |
| YGMCC0102 | *Bifidobacterium longum* | 2.417 | *B.longum* | *B.longum* | *B.longum* | *B.longum* | *B.longum* |
| YGMCC0458 | *Bifidobacterium longum* | 2.363 | *B.longum* | *B.longum* | *B.longum* | *B.longum* | *B.longum* |
| YGMCC0302 | *Bifidobacterium longum* | 2.557 | *B.longum* | *B.longum* | *B.longum* | *B.longum* | *B.longum* |
| YGMCC0830 | *Bifidobacterium longum* | 2.534 | *B.longum* | *B.longum* | *B.longum* | *B.longum* | *B.longum* |
| YGMCC0721 | *Bifidobacterium longum* | 2.516 | *B.longum* | *B.longum* | *B.longum* | *B.longum* | *B.longum* |
| YGMCC0446 | *Bifidobacterium longum* | 2.493 | *B.longum* | *B.longum* | *B.longum* | *B.longum* | *B.longum* |
| YGMCC0410 | *Bifidobacterium longum* | 2.593 | *B.longum* | *B.longum* | *B.longum* | *B.longum* | *B.longum* |
| YGMCC0870 | *Bifidobacterium longum* | 2.600 | *B.longum* | *B.longum* | *B.longum* | *B.longum* | *B.longum* |
| YGMCC0038 | *Bifidobacterium longum* | 2.352 | *B.longum* | *B.longum* | *B.longum* | *B.infantis* | *B.longum* |
| YGMCC0049 | *Bifidobacterium longum* | 2.440 | *B.longum* | *B.longum* | *B.longum* | *B.longum* | *B.longum* |
| YGMCC0615 | *Bifidobacterium longum* | 2.400 | *B.longum* | *B.longum* | *B.longum* | *B.longum* | *B.longum* |
| YGMCC0784 | *Bifidobacterium longum* | 2.304 | *B.longum* | *B.longum* | *B.longum* | *B.longum* | *B.longum* |
| YGMCC0406 | *Bifidobacterium longum* | 2.525 | *B.longum* | *B.longum* | *B.longum* | *B.longum* | *B.longum* |
| YGMCC0325 | *Bifidobacterium longum* | 2.582 | *B.infantis* | *B.infantis* | *B.infantis* | *B.infantis* | *B.infantis* |
| YGMCC0484 | *Bifidobacterium longum* | 2.391 | *B.infantis* | *B.infantis* | *B.infantis* | *B.infantis* | *B.infantis* |
| YGMCC0469 | *Bifidobacterium longum* | 2.518 | *B.infantis* | *B.infantis* | *B.infantis* | *B.infantis* | *B.infantis* |
| YGMCC0935 | *Bifidobacterium longum* | 2.370 | *B.infantis* | *B.infantis* | *B.infantis* | *B.infantis* | *B.infantis* |
| YGMCC0538 | *Bifidobacterium longum* | 2.492 | *B.infantis* | *B.infantis* | *B.infantis* | *B.infantis* | *B.infantis* |
| YGMCC0483 | *Bifidobacterium longum* | 2.322 | *B.infantis* | *B.infantis* | *B.infantis* | *B.infantis* | *B.infantis* |
| YGMCC0192 | *Bifidobacterium longum* | 2.530 | *B.infantis* | *B.infantis* | *B.longum* | *B.infantis* | *B.infantis* |
| YGMCC0429 | *Bifidobacterium longum* | 2.516 | *B.infantis* | *B.infantis* | *B.infantis* | *B.infantis* | *B.infantis* |
| YGMCC0904 | *Bifidobacterium longum* | 2.644 | *B.infantis* | *B.infantis* | *B.infantis* | *B.infantis* | *B.infantis* |
| YGMCC0271 | *Bifidobacterium longum* | 2.367 | *B.infantis* | *B.infantis* | *B.longum* | *B.infantis* | *B.infantis* |
| YGMCC0005 | *Bifidobacterium longum* | 2.565 | *B.infantis* | *B.infantis* | *B.infantis* | *B.infantis* | *B.infantis* |
| YGMCC0507 | *Bifidobacterium longum* | 2.491 | *B.infantis* | *B.infantis* | *B.infantis* | *B.infantis* | *B.infantis* |
| YGMCC0922 | *Bifidobacterium longum* | 2.407 | *B.infantis* | *B.infantis* | *B.infantis* | *B.infantis* | *B.infantis* |
| YGMCC0069 | *Bifidobacterium longum* | 2.505 | *B.infantis* | *B.infantis* | *B.infantis* | *B.infantis* | *B.infantis* |
| YGMCC0593 | *Bifidobacterium longum* | 2.389 | *B.infantis* | *B.infantis* | *B.infantis* | *B.infantis* | *B.infantis* |
| YGMCC0669 | *Bifidobacterium longum* | 2.476 | *B.infantis* | *B.infantis* | *B.infantis* | *B.infantis* | *B.infantis* |
| YGMCC0227 | *Bifidobacterium longum* | 2.338 | *B.infantis* | *B.infantis* | *B.infantis* | *B.infantis* | *B.infantis* |
| YGMCC0040 | *Bifidobacterium longum* | 2.342 | *B.infantis* | *B.infantis* | *B.infantis* | *B.infantis* | *B.infantis* |
| YGMCC0020 | *Bifidobacterium longum* | 2.598 | *B.infantis* | *B.infantis* | *B.infantis* | *B.infantis* | *B.infantis* |
| YGMCC0550 | *Bifidobacterium longum* | 2.350 | *B.infantis* | *B.infantis* | *B.longum* | *B.infantis* | *B.infantis* |
| YGMCC0556 | *Bifidobacterium longum* | 2.426 | *B.infantis* | *B.infantis* | *B.infantis* | *B.infantis* | *B.infantis* |
| YGMCC0233 | *Bifidobacterium longum* | 2.473 | *B.infantis* | *B.infantis* | *B.infantis* | *B.infantis* | *B.infantis* |
| YGMCC0295 | *Bifidobacterium longum* | 2.457 | *B.infantis* | *B.infantis* | *B.infantis* | *B.infantis* | *B.infantis* |
| YGMCC0653 | *Bifidobacterium longum* | 2.388 | *B.infantis* | *B.infantis* | *B.infantis* | *B.infantis* | *B.infantis* |
| YGMCC0311 | *Bifidobacterium longum* | 2.465 | *B.infantis* | *B.infantis* | *B.infantis* | *B.infantis* | *B.infantis* |
| YGMCC0120 | *Bifidobacterium longum* | 2.533 | *B.infantis* | *B.infantis* | *B.infantis* | *B.infantis* | *B.longum* |
| YGMCC0110 | *Bifidobacterium longum* | 2.546 | *B.infantis* | *B.infantis* | *B.infantis* | *B.infantis* | *B.infantis* |
| YGMCC0643 | *Bifidobacterium longum* | 2.326 | *B.infantis* | *B.infantis* | *B.infantis* | *B.infantis* | *B.infantis* |
| YGMCC0145 | *Bifidobacterium longum* | 2.317 | *B.infantis* | *B.infantis* | *B.infantis* | *B.infantis* | *B.infantis* |
| YGMCC0494 | *Bifidobacterium longum* | 2.328 | *B.infantis* | *B.infantis* | *B.infantis* | *B.infantis* | *B.infantis* |
| YGMCC0146 | *Bifidobacterium longum* | 2.497 | *B.longum* | *B.longum* | *——* | *——* | *——* |
| YGMCC0230 | *Bifidobacterium longum* | 2.433 | *B.longum* | *B.longum* | *——* | *——* | *——* |
| YGMCC0190 | *Bifidobacterium longum* | 2.587 | *B.longum* | *B.longum* | *——* | *——* | *——* |
| YGMCC0209 | *Bifidobacterium longum* | 2.521 | *B.longum* | *B.longum* | *——* | *——* | *——* |
| YGMCC0297 | *Bifidobacterium longum* | 2.369 | *B.longum* | *B.longum* | *——* | *——* | *——* |
| YGMCC0039 | *Bifidobacterium longum* | 2.496 | *B.longum* | *B.longum* | *——* | *——* | *——* |
| YGMCC0057 | *Bifidobacterium longum* | 2.565 | *B.longum* | *B.longum* | *——* | *——* | *——* |
| YGMCC0091 | *Bifidobacterium longum* | 2.372 | *B.longum* | *B.longum* | *——* | *——* | *——* |
| YGMCC0335 | *Bifidobacterium longum* | 2.530 | *B.longum* | *B.longum* | *——* | *——* | *——* |
| YGMCC0308 | *Bifidobacterium longum* | 2.307 | *B.longum* | *B.longum* | *——* | *——* | *——* |
| YGMCC0718 | *Bifidobacterium longum* | 2.404 | *B.longum* | *B.longum* | *——* | *——* | *——* |
| YGMCC0514 | *Bifidobacterium longum* | 2.415 | *B.longum* | *B.longum* | *——* | *——* | *——* |
| YGMCC0767 | *Bifidobacterium longum* | 2.302 | *B.longum* | *B.longum* | *——* | *——* | *——* |
| YGMCC0478 | *Bifidobacterium longum* | 2.551 | *B.longum* | *B.longum* | *——* | *——* | *——* |
| YGMCC0341 | *Bifidobacterium longum* | 2.357 | *B.longum* | *B.longum* | *——* | *——* | *——* |
| YGMCC0388 | *Bifidobacterium longum* | 2.550 | *B.longum* | *B.longum* | *——* | *——* | *——* |
| YGMCC0051 | *Bifidobacterium longum* | 2.341 | *B.longum* | *B.longum* | *——* | *——* | *——* |
| YGMCC0524 | *Bifidobacterium longum* | 2.429 | *B.longum* | *B.longum* | *——* | *——* | *——* |
| YGMCC0008 | *Bifidobacterium longum* | 2.411 | *B.longum* | *B.longum* | *——* | *——* | *——* |
| YGMCC0619 | *Bifidobacterium longum* | 2.365 | *B.longum* | *B.longum* | *——* | *——* | *——* |
| YGMCC0650 | *Bifidobacterium longum* | 2.412 | *B.longum* | *B.longum* | *——* | *——* | *——* |
| YGMCC0919 | *Bifidobacterium longum* | 2.582 | *B.longum* | *B.longum* | *——* | *——* | *——* |
| YGMCC0831 | *Bifidobacterium longum* | 2.355 | *B.longum* | *B.longum* | *——* | *——* | *——* |
| YGMCC0327 | *Bifidobacterium longum* | 2.352 | *B.longum* | *B.longum* | *——* | *——* | *——* |
| YGMCC0633 | *Bifidobacterium longum* | 2.340 | *B.longum* | *B.longum* | *——* | *——* | *——* |
| YGMCC0626 | *Bifidobacterium longum* | 2.343 | *B.longum* | *B.longum* | *——* | *——* | *——* |
| YGMCC0134 | *Bifidobacterium longum* | 2.547 | *B.longum* | *B.longum* | *——* | *——* | *——* |
| YGMCC0225 | *Bifidobacterium longum* | 2.366 | *B.longum* | *B.longum* | *——* | *——* | *——* |
| YGMCC0533 | *Bifidobacterium longum* | 2.373 | *B.longum* | *B.longum* | *——* | *——* | *——* |
| YGMCC0838 | *Bifidobacterium longum* | 2.352 | *B.longum* | *B.longum* | *——* | *——* | *——* |
| YGMCC0983 | *Bifidobacterium longum* | 2.394 | *B.longum* | *B.longum* | *——* | *——* | *——* |
| YGMCC0172 | *Bifidobacterium longum* | 2.543 | *B.longum* | *B.longum* | *——* | *——* | *——* |
| YGMCC0358 | *Bifidobacterium longum* | 2.397 | *B.longum* | *B.longum* | *——* | *——* | *——* |
| YGMCC0689 | *Bifidobacterium longum* | 2.336 | *B.longum* | *B.longum* | *——* | *——* | *——* |
| YGMCC0200 | *Bifidobacterium longum* | 2.402 | *B.longum* | *B.longum* | *——* | *——* | *——* |
| YGMCC0552 | *Bifidobacterium longum* | 2.594 | *B.longum* | *B.longum* | *——* | *——* | *——* |
| YGMCC0391 | *Bifidobacterium longum* | 2.595 | *B.longum* | *B.longum* | *——* | *——* | *——* |
| YGMCC0888 | *Bifidobacterium longum* | 2.325 | *B.longum* | *B.longum* | *——* | *——* | *——* |
| YGMCC0783 | *Bifidobacterium longum* | 2.498 | *B.longum* | *B.longum* | *——* | *——* | *——* |
| YGMCC0606 | *Bifidobacterium longum* | 2.387 | *B.longum* | *B.longum* | *——* | *——* | *——* |
| YGMCC0850 | *Bifidobacterium longum* | 2.566 | *B.longum* | *B.longum* | *——* | *——* | *——* |
| YGMCC0355 | *Bifidobacterium longum* | 2.461 | *B.longum* | *B.longum* | *——* | *——* | *——* |
| YGMCC0157 | *Bifidobacterium longum* | 2.315 | *B.longum* | *B.longum* | *——* | *——* | *——* |
| YGMCC0536 | *Bifidobacterium longum* | 2.344 | *B.longum* | *B.longum* | *——* | *——* | *——* |
| YGMCC0027 | *Bifidobacterium longum* | 2.586 | *B.longum* | *B.longum* | *——* | *——* | *——* |
| YGMCC0955 | *Bifidobacterium longum* | 2.569 | *B.longum* | *B.longum* | *——* | *——* | *——* |
| YGMCC0757 | *Bifidobacterium longum* | 2.547 | *B.longum* | *B.longum* | *——* | *——* | *——* |
| YGMCC0062 | *Bifidobacterium longum* | 2.512 | *B.longum* | *B.longum* | *——* | *——* | *——* |
| YGMCC0220 | *Bifidobacterium longum* | 2.316 | *B.longum* | *B.longum* | *——* | *——* | *——* |
| YGMCC0195 | *Bifidobacterium longum* | 2.403 | *B.longum* | *B.longum* | *——* | *——* | *——* |
| YGMCC0827 | *Bifidobacterium longum* | 2.374 | *B.longum* | *B.longum* | *——* | *——* | *——* |
| YGMCC0720 | *Bifidobacterium longum* | 2.492 | *B.longum* | *B.longum* | *——* | *——* | *——* |
| YGMCC0315 | *Bifidobacterium longum* | 2.319 | *B.longum* | *B.longum* | *——* | *——* | *——* |
| YGMCC0180 | *Bifidobacterium longum* | 2.351 | *B.longum* | *B.longum* | *——* | *——* | *——* |
| YGMCC0025 | *Bifidobacterium longum* | 2.499 | *B.infantis* | *B.infantis* | *——* | *——* | *——* |
| YGMCC0979 | *Bifidobacterium longum* | 2.574 | *B.infantis* | *B.infantis* | *——* | *——* | *——* |
| YGMCC0846 | *Bifidobacterium longum* | 2.501 | *B.infantis* | *B.infantis* | *——* | *——* | *——* |
| YGMCC0770 | *Bifidobacterium longum* | 2.518 | *B.infantis* | *B.infantis* | *——* | *——* | *——* |
| YGMCC0555 | *Bifidobacterium longum* | 2.353 | *B.infantis* | *B.infantis* | *——* | *——* | *——* |
| YGMCC0735 | *Bifidobacterium longum* | 2.557 | *B.infantis* | *B.infantis* | *——* | *——* | *——* |
| YGMCC0516 | *Bifidobacterium longum* | 2.484 | *B.infantis* | *B.infantis* | *——* | *——* | *——* |
| YGMCC0186 | *Bifidobacterium longum* | 2.548 | *B.infantis* | *B.infantis* | *——* | *——* | *——* |
| YGMCC0685 | *Bifidobacterium longum* | 2.518 | *B.infantis* | *B.infantis* | *——* | *——* | *——* |
| YGMCC0978 | *Bifidobacterium longum* | 2.449 | *B.infantis* | *B.infantis* | *——* | *——* | *——* |
| YGMCC0969 | *Bifidobacterium longum* | 2.336 | *B.infantis* | *B.infantis* | *——* | *——* | *——* |
| YGMCC0088 | *Bifidobacterium longum* | 2.446 | *B.infantis* | *B.infantis* | *——* | *——* | *——* |
| YGMCC0759 | *Bifidobacterium longum* | 2.333 | *B.infantis* | *B.infantis* | *——* | *——* | *——* |
| YGMCC0436 | *Bifidobacterium longum* | 2.484 | *B.infantis* | *B.infantis* | *——* | *——* | *——* |
| YGMCC0491 | *Bifidobacterium longum* | 2.423 | *B.infantis* | *B.infantis* | *——* | *——* | *——* |
| YGMCC0746 | *Bifidobacterium longum* | 2.309 | *B.infantis* | *B.infantis* | *——* | *——* | *——* |
| YGMCC0938 | *Bifidobacterium longum* | 2.300 | *B.infantis* | *B.infantis* | *——* | *——* | *——* |
| YGMCC0728 | *Bifidobacterium longum* | 2.483 | *B.infantis* | *B.infantis* | *——* | *——* | *——* |
| YGMCC0794 | *Bifidobacterium longum* | 2.320 | *B.infantis* | *B.infantis* | *——* | *——* | *——* |
| YGMCC0181 | *Bifidobacterium longum* | 2.533 | *B.infantis* | *B.infantis* | *——* | *——* | *——* |
| YGMCC0480 | *Bifidobacterium longum* | 2.366 | *B.infantis* | *B.infantis* | *——* | *——* | *——* |
| YGMCC0672 | *Bifidobacterium longum* | 2.571 | *B.infantis* | *B.infantis* | *——* | *——* | *——* |
| YGMCC0609 | *Bifidobacterium longum* | 2.426 | *B.infantis* | *B.infantis* | *——* | *——* | *——* |
| YGMCC0092 | *Bifidobacterium longum* | 2.552 | *B.infantis* | *B.infantis* | *——* | *——* | *——* |
| YGMCC0644 | *Bifidobacterium longum* | 2.538 | *B.infantis* | *B.infantis* | *——* | *——* | *——* |
| YGMCC0472 | *Bifidobacterium longum* | 2.369 | *B.infantis* | *B.infantis* | *——* | *——* | *——* |
| YGMCC0490 | *Bifidobacterium longum* | 2.451 | *B.infantis* | *B.infantis* | *——* | *——* | *——* |
| YGMCC0574 | *Bifidobacterium longum* | 2.332 | *B.infantis* | *B.infantis* | *——* | *——* | *——* |
| YGMCC0918 | *Bifidobacterium longum* | 2.590 | *B.infantis* | *B.infantis* | *——* | *——* | *——* |
| YGMCC0456 | *Bifidobacterium longum* | 2.501 | *B.infantis* | *B.infantis* | *——* | *——* | *——* |
| YGMCC0194 | *Bifidobacterium longum* | 2.549 | *B.infantis* | *B.infantis* | *——* | *——* | *——* |
| YGMCC0054 | *Bifidobacterium longum* | 2.521 | *B.infantis* | *B.infantis* | *——* | *——* | *——* |
| YGMCC0167 | *Bifidobacterium longum* | 2.548 | *B.infantis* | *B.infantis* | *——* | *——* | *——* |
| YGMCC0868 | *Bifidobacterium longum* | 2.520 | *B.infantis* | *B.infantis* | *——* | *——* | *——* |
| YGMCC0586 | *Bifidobacterium longum* | 2.407 | *B.infantis* | *B.infantis* | *——* | *——* | *——* |

The red and blue background represent internal and external dataset, respectively.
